# Isolation and characterization of ovine mesenchymal stem cells derived from peripheral blood

**DOI:** 10.1186/1746-6148-8-169

**Published:** 2012-09-22

**Authors:** Jaber Lyahyai, Diego R Mediano, Beatriz Ranera, Arianne Sanz, Ana Rosa Remacha, Rosa Bolea, Pilar Zaragoza, Clementina Rodellar, Inmaculada Martín-Burriel

**Affiliations:** 1 Laboratorio de Genética Bioquímica (LAGENBIO), Facultad de Veterinaria, Universidad de Zaragoza, Miguel Servet 177, Zaragoza, 50013, Spain; 2 Centre de Génomique Humaine, Faculté de Médecine et Pharmacie, Université Mohammed V Souissi, Rabat, Morocco; 3 Centro de Investigación en Encefalopatías y Enfermedades Transmisibles Emergentes (CIEETE), Facultad de Veterinaria, Universidad de Zaragoza, Zaragoza, Spain; 4 Instituto Aragonés de Ciencias de la Salud, Zaragoza, Spain

**Keywords:** Sheep, Mesenchymal stem cell, Peripheral blood, Neurogenesis

## Abstract

**Background:**

Mesenchymal stem cells (MSCs) are multipotent stem cells with capacity to differentiate into several mesenchymal lineages. This quality makes MSCs good candidates for use in cell therapy. MSCs can be isolated from a variety of tissues including bone marrow and adipose tissue, which are the most common sources of these cells. However, MSCs can also be isolated from peripheral blood. Sheep has been proposed as an ideal model for biomedical studies including those of orthopaedics and transmissible spongiform encephalopathies (TSEs). The aim of this work was to advance these studies by investigating the possibility of MSC isolation from ovine peripheral blood (oPB-MSCs) and by subsequently characterizing there *in vitro* properties.

**Results:**

Plastic-adherent fibroblast-like cells were obtained from the mononuclear fraction of blood samples. These cells were analysed for their proliferative and differentiation potential into adipocytes, osteoblasts and chondrocytes, as well as for the gene expression of cell surface markers. The isolated cells expressed transcripts for markers *CD29*, *CD73* and *CD90*, but failed to express the haematopoietic marker *CD45* and expressed only low levels of *CD105*. The expression of *CD34* was variable. The differentiation potential of this cell population was evaluated using specific differentiation media. Although the ability of the cultures derived from different animals to differentiate into adipocytes, osteoblasts and chondrocytes was heterogeneous, we confirmed this feature using specific staining and analysing the gene expression of differentiation markers. Finally, we tested the ability of oPB-MSCs to transdifferentiate into neuronal-like cells. Morphological changes were observed after 24-hour culture in neurogenic media, and the transcript levels of the neurogenic markers increased during the prolonged induction period. Moreover, oPB-MSCs expressed the cellular prion protein gene (*PRNP*), which was up-regulated during neurogenesis.

**Conclusions:**

This study describes for the first time the isolation and characterization of oPB-MSCs. Albeit some variability was observed between animals, these cells retained their capacity to differentiate into mesenchymal lineages and to transdifferentiate into neuron-like cells *in vitro***.** Therefore, oPB-MSCs could serve as a valuable tool for biomedical research in fields including orthopaedics or prion diseases.

## Background

Mesenchymal stem cells (MSCs) are morphologically fibroblast-like cells that are characterized by their ability to both self-renew and differentiate into tissues of mesodermal origin (osteoblasts, adipocytes, chondrocytes and myocytes) [1]. However, MSCs can also give rise to other cell types such as astrocytes and neurons [2,3]. This indicates cellular pluripotency and suggests that MSCs are responsible for the normal turnover and maintenance of adult mesenchymal tissues [4].

Sheep is an ideal model for bone tissue engineering [5] and has been proposed as an animal model for a wide range of applications in biomedical research, such as for the studies of respiratory diseases [6], cardiomyopathies [7,8], neurological disorders [9] and prion diseases [10,11].

Although MSCs are generally obtained from the bone marrow [12], they can also be isolated from other sources such as adipose tissue, umbilical cord blood and foetal tissues [13,14]. The isolation of MSCs from peripheral blood (PB-MSCs) has been reported for a variety of mammals including guinea pigs, rabbits, dogs, mice, rats, horses and humans [15-19]. Because blood harvesting is a less invasive procedure to obtain stem cells, this method would represent a significant advantage for patients and, therefore, would be an ideal candidate technique to obtain PB-MSCs for future clinical applications. Moreover, monitoring the presence and the proportional quantity of MSCs in the peripheral blood could possibly help in the understanding of the patients’ reaction to a disease.

The isolation procedure of ovine PB-MSCs (oPB-MSCs) would facilitate the sampling of these progenitor cells for use in a wide variety of applications, including fundamental and applied studies of orthopaedics or prion diseases. Here, we present the first study describing the isolation and characterization of oPB-MSCs. The osteogenic, chondrogenic and adipogenic differentiation potential of oPB-MSCs was analysed *in vitro* and monitored by specific staining and molecular differentiation markers. We also demonstrate the capacity of these cells to differentiate into neuron-like cells and the expression of the gene coding for the prion protein (*PRNP*) in both regular and differentiated cells.

## Results

### Isolation and characterization of peripheral blood derived fibroblast-like cells

#### Isolation and expansion of peripheral blood derived fibroblast-like cells

Plastic-adherent fibroblast-like cells were observed within the first days of culture of the nucleated cell fraction of peripheral blood obtained from total six sheep. Although the volume of blood collected was similar for all animals (approximately 25 mL), the number of peripheral blood nucleated cells (PBNC) obtained was variable, ranging from 0.594 10^6^ to 1.9 10^6^ PBNC/mL, with mean 1.36 10^6^ ± 682646. After the isolation process, a mean of 281400 ± 178051 adherent cells were obtained from each individual, varying between 2.7 and 9.3 adherent cells for every 1000 PBNC (mean: 5.85 ± 2.7).

Cells were expanded until the second passage and then frozen. The proliferation capacity of the adherent cells was measured during the first two passages. An average of 12.6 days was necessary to complete the first passage. Mean cell doubling during the first passage was 2.29 ± 0.887 and the doubling time was 5.99 ± 1.86 days. Time required to complete the second passage was shortened to 7.33 days, cell doubling decreased to 1.84 ± 0.975 and the doubling time was 4.88 ± 2.68 days.

After thawing, the cells from passage 2 were expanded for two more passages to obtain sufficient amount of cells for the differentiation assays. The cells were then characterized by analysing the expression of cell surface markers and the tri-lineage differentiation potential into adipocytes, osteoblasts and chondrocytes.

#### Expression of mesenchymal cell surface markers

To initiate the characterization of oPB-MSCs, the expression of six cell surface markers specific for mesenchymal and haematopoietic cells were first analysed at the transcript level by quantitative real time PCR (RT-qPCR). All analysed cultures expressed *CD29* (integrin β1), *CD73* (ecto-5’-nucleotidase) and *CD90* (Thy-1), whereas the expression of *CD34* (CD34 molecule) was detected in five out of six of these cultures. The amplification of the hematopoietic marker *CD45* (protein tyrosine phosphatase, receptor type, C) was not detected and *CD105* (endoglin) was only weakly amplified at threshold cycles above 35.

#### Adipogenic potential

**Figure 1:**
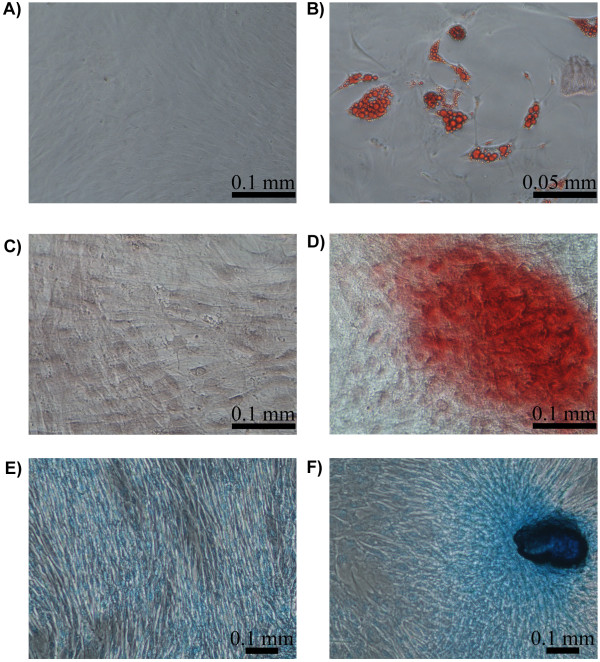
**Staining for adipogenic, osteogenic and chondrogenic differentiation of PB-MSCs.** Oil red O staining of cells cultured for 15 days in basal (**A**) and adipogenic differentiation medium (**B**). Alizarin red staining of cells cultured for 21 days in basal (**C)** and osteogenic differentiation medium (**D**). Alcian blue staining of cells cultured for 21 days in basal (**E**) and chondrogenic medium (**F**).

**Figure 2:**
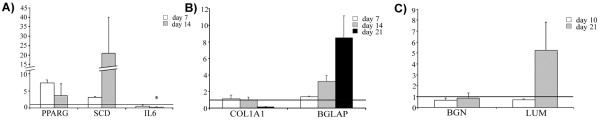
**Quantitative real time PCR analysis.** Expression of the adipogenic (**A**), osteogenic (**B**) and chondrogenic (**C**) markers at different times of the differentiation process relative to the levels observed in control cultures (values set to 1, horizontal line)**.** Data are shown as mean ± standard errors. Statistically significant differences between differentiated and control cells were determined by Student t test (*P < 0.05).

Cells cultured under adipogenic conditions presented cytoplasmic lipid droplets under light microscope, although the size of the droplets was variable depending on the donor animal. To confirm that the contents of the droplets were lipids, the cultures were stained with oil red O (Figure 1A and B). The expression of adipogenic markers was analysed on days 7 and 14 of post-induction. The expression profiles of *PPARG* (peroxisome proliferator-activated receptor gamma), *SCD* (stearoyl-CoA desaturase) and *IL6* (interleukin 6) are shown in Figure 2. During the induction of differentiation, the *PPARG* and *SCD* mRNA expression levels increased to 7.3- and 20.8-fold, respectively. However, these changes were not statistically significant due to the high variability observed between animals. A significant downregulation of *IL6* (−31-fold, *P* < 0.05) was detected after two weeks of culture (Figure 2A).

#### Osteogenic potential

The ability of oPB-MSCs to differentiate into osteoblasts was demonstrated using alizarin red staining (Figure 1C and D). Nodule-like aggregations stained in red appeared in the osteogenic media on the 21^st^ day of culture exclusively, indicating that these cultures were mineralized at a relatively late stage. However, the cells from different animals displayed variable osteogenic potential. The expression of osteogenic markers was evaluated in the cultures that displayed positive staining (n = 2). The expression levels of *COL1A1* (collagen, type 1, α 1) were not altered during the first 2 weeks in osteogenic media. However, a strong downregulation of *COL1A1* was observed at 3 weeks of culture. In contrast, the expression levels of *BGLAP* (bone gamma-carboxyglutamate (gla) protein, or osteocalcin) increased drastically throughout the culture period (Figure 2B).

#### Chondrogenic potential

The chondrogenic potential was evaluated in monolayer cultures. Ovine PB-MSCs formed nodule-like aggregations in both control and induced conditions. However, the oPB-MSCs in chondrogenic media displayed a stronger staining with alcian blue (Figure 1F). Although the chondrogenic marker expression analysis did not reveal variations in the gene expression levels of the *BGN* (biglycan), *LUM* (lumican) was found to be upregulated on the 21^st^ day of culture (Figure 2C).

### Neuronal differentiation of oPB-MSCs

**Figure 3:**
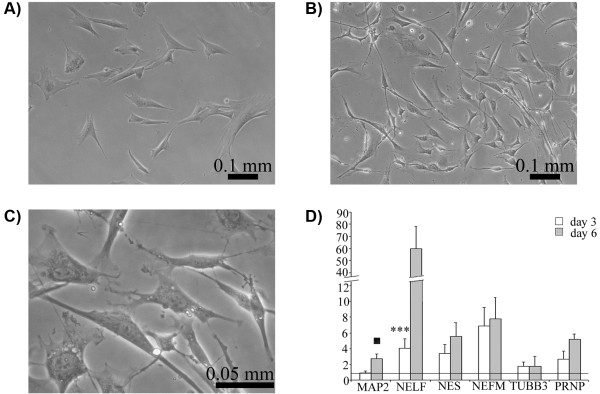
**Neurogenic differentiation of PB-MSCs.** Phase contrast micrograph of ovine PB-MSCs at passage 3 cultured on basal and neurogenic medium for 3 days. Control cells (**A**) showed a fibroblast-like shape whereas differentiated cells (**B**) displayed neuronal-like features such as phase-bright bodies, long multipolar extensions and branching ends (**C**: higher magnification). Increase of the expression levels of neurogenic markers after 3 (white bars) and 6 (grey bars) days of induction relative to the levels observed in cells cultured on basal medium (values set to 1, horizontal line) as assessed by real time PCR. Data are shown as mean ± standard errors. Statistically significant differences between differentiated and control cells were determined by Student t test (▪ *P* < 0.10, *** *P* < 0.001).

The ability of the isolated cells to transdifferentiate into neuronal cells was evaluated *in vitro*. The cells cultured under neurogenic conditions displayed distinctly altered morphology after the first 24 hours of induction. Differentiated cells were sharply defined, retracted towards the nucleus displaying phase-bright bodies, and some neurite-like processes (thin, long, and often branched) became apparent (Figure 3B,C). Neuronal differentiation was also demonstrated using RT-qPCR analysis. Control cells displayed none or very low levels of *NELF* (nasal embryonic LHRH factor) expression on 3 and 6 days of culture, while low expression levels of the remaining markers (*MAP2* [microtubule-associated protein 2], *NES* [nestin], *NEFM* [neurofilament, medium polypeptide], *TUBB3* [tubulin, beta 3]) were observed. The expression of these markers increased in neurogenic conditions, with a peak of expression on day 6 post-induction. Statistically significant changes were found for *NELF* on day 3 of culture (5.85 fold induction, *P* < 0.001) and an over-expression tendency was observed for *MAP2* on day 6 (2.4 fold induction, *P* < 0.1). Moreover, oPB-MSCs expressed transcripts of the prion protein (*PRNP*), which increased up to 5 times during the neurogenic period (Figure 3D).

## Discussion

Despite the importance of ovine as a large animal model for many conditions (i.e., orthopaedic injuries or Transmissible Spongiform Encephalopathies) the characterisation of ovine MSCs (oMSCs) is still limited. During the last decade, there has been an important effort within the scientific community to focus on the characterisation of MSCs obtained from different species, including the sheep. However, most research on MSCs has been performed on cells derived from bone marrow and, to a lesser degree, adipose tissue. The osteogenic and chondrogenic differentiation potential of MSCs *in vitro*[20-22] and *in vivo*[23,24] is currently relatively well understood. Their phenotype for mesenchymal surface cell markers has also been analysed [25], and their proliferative potential has been shown to be heterogeneous [26]. Although the existence of MSCs in peripheral blood has been demonstrated in many species [17,18], this work represents the first report describing the isolation of these cells from sheep circulation.

The minimal criteria to define human MSCs proposed by the Mesenchymal and Tissue Stem Cell Committee of the International Society for Cellular Therapy are: (1) plastic-adhesion when maintained in standard culture conditions; (2) expression of CD105, CD73 and CD90, and lack expression of the haematopoietic markers CD45, CD34, CD14 or CD11b, CD79alpha or CD19 and HLA-DR surface molecules and; (3) ability to differentiate to osteoblasts, adipocytes and chondroblasts *in vitro*[27]. In our study, plastic-adherent cells with a fibroblast-like morphology were obtained from all experimental sheep and were further analysed to determine the expression of mesenchymal markers and their ability to differentiate into adipocytes, osteoblasts and chondrocytes.

In other domestic species [28] the proportion of MSCs in the peripheral blood is low, which is in agreement with the few colonies of MSCs detected in our original oPB-MSCs cultures. Although the proliferation ability of oPB-MSCs was very different between individuals, the doubling time was generally longer than in other species, such as horse [29]. This difference may be due to the higher percentage of FBS used in the isolation of equine PB-MSCs (30%) and also due to the addition of dexamethasone to the growth media, which has been demonstrated to favour the expansion of MSCs [30]. The variability observed in this work is in accordance with the high heterogeneity in the proliferative potential of oMSCs obtained from bone marrow (oBM-MSCs) [26].

The absence of a well-defined immunophenotype for PB-MSCs renders the comparison of studies difficult. Moreover, most of the cell surface markers utilized to sort subpopulations of human MSC by flow cytometry have not been validated in sheep [21]. Gene expression-based technologies may be useful for the identification of possible molecules described as MSC markers [31,32]. In our study, RT-qPCR was performed to quantify the mRNA expression levels of six cell surface antigens considered as either positive (*CD29*, *CD73*, *CD90* and *CD105*) or negative (*CD34* and *CD45*) MSC markers in humans.

In accordance with the immunophenotype described for human PB-MSCs [33-35], our expression analysis revealed significant amplification of the typical MSC markers, *CD29*, *CD73* and *CD90*, and a weak signal for *CD105*. In contrast, the haematopoietic marker *CD45* was not expressed. To our knowledge, there are no published data concerning the gene expression of cell surface markers in oMSCs obtained from other tissues. However, we have observed amplification of *CD29*, *CD73* and *CD90* in oBM-MSCs, as well as the lack of *CD34* and *CD45* expression (unpublished work from our group). Using flow cytometry, the presence of CD29 and CD105 has also been detected in oBM-MSCs [21,36]. Additionally, oMSCs isolated from adipose tissue (oAT-MSCs) display high expression of CD90 and low immunoreactivity for CD105 [25]. The immunophenotypes of oBM-MSCs [36] and oAT-MSCs [25] are negative for the haematopoietic CD34 marker. However, this marker is expressed at low levels in human PB-MSCs [37] and in equine MSCs derived from adipose tissue [38] as demonstrated by RT-qPCR. We detected *CD34* expression in 5 out of 6 cultures, which may indicate individual variability. Finally, the cells analysed were negative for the haematopoietic marker *CD45*, as are human MSCs [16]. We have previously found a good correlation between MSC marker gene expression and the immunophenotype detected by flow cytometry in equine MSCs [39]. Although flow cytometry analysis is necessary to validate the immunophenotype of the isolated cells, the gene expression profile observed in this work strongly suggests that the peripheral blood derived fibroblast-like cells obtained as described would fulfill the requirements to be considered as MSCs.

Ovine BM-MSCs can be differentiated into adipocytes, showing lipid droplets in their cytoplasm and the induction of adipogenic markers [20,36]. Similarly, adipogenic differentiation has been achieved here in all peripheral blood derived cell cultures, although great variability in the size of lipid droplets was observed. The expression of two adipogenic markers was evaluated in the cultures using RT-qPCR. PPARG is considered the master regulator of adipogenesis [40,41] and is up-regulated in MSCs under adipogenic conditions [42]. SCD is expressed uniquely in adipocytes and catalyzes the rate-limiting step in the synthesis of poly-unsaturated fatty acids, thereby exhibiting a pivotal role in adipocyte metabolism [43]. Inter-individual variability was also noticeable in the expression of these adipogenic markers, which explains the lack of statistically significant differences in *PPARG* and *SCD* expression results despite the strong overexpression observed throughout the culture period. We also determined the expression of IL-6, which maintains the proliferative and undifferentiated state of bone marrow-derived MSCs [44] and is down-regulated during lineage-specific differentiation [45]. In accordance with these reports, a significant decrease was detected in the expression of *IL6* in the differentiated cultures. Therefore, using specific staining and gene expression profiles of adipogenic markers, we have confirmed the adipogenic potential of oPB-MSCs.

Similar to the adipogenic analysis, a great individual variation was also observed in the osteogenic potential. Osteogenic mineralization was confirmed on the last day of culture in osteogenic conditions (21 days) by staining calcium deposits with alizarin red. The induction period necessary for visualization of matrix mineralisation in oMSCs varies among different studies. The period reported for oBM-MSC mineralisation ranges from 21 days [36] to 5 weeks [21], while 4 weeks are required to differentiate periodontal oMSCs [46]. The weak alizarin red staining observed in some of our experiments could be due to the relatively short period of induction.

Although COL1A1 is considered an early marker of osteoprogenitor cells [47], we observed either no changes or a strong down-regulation on the 21st day of culture. Besides displaying a rapid mineralisation, oBM-MSCs cultured under osteogenic conditions express increased or declined levels of COL1A1 depending on the differentiation moment [21]. Other authors have reported either no significant increase in *COL1A1* mRNA expression levels after osteogenic differentiation in human [48], porcine [49] and equine [39] MSCs, or a down-regulation of this marker in human PB-MSCs during osteogenesis [50]. Therefore, *COL1A1* may not be suitable for monitoring osteogenesis in oPB-MSCs. In contrast, *BGLAP* was upregulated during the differentiation process and was maximally expressed on the last day of culture (day 21), coinciding with the positive alizarin red staining. This is in accordance with the role of BGLAP as a late marker of developing osteoblasts [51].

The sheep has been used as a large animal model for the studies of chondrogenesis both *in vivo*[52] and *in vitro*[22]. The chondrogenic potential of oMSCs has been evaluated mainly in micromass cultures of cells derived from bone marrow [20,21,36]. Chondrogenesis was evaluated in our study using a bidimensional culture with a high cell concentration seeding, according to the protocol described by Jäger et al. [53] for chondrogenic differentiation of ovine umbilical cord blood-derived MSCs. Chondrogenic nodules were observed in both control and chondrogenic media, although the staining was stronger in the induced cultures. The confirmation with molecular markers was not straightforward as the expression of the two components of the extracellular matrix *BGN* and *LUM* changed in opposite directions during chondrogenic differentiation. In accordance to our results, the lack of strong *BGN* overexpression has been reported for chondrogenic induced micropellets of oBM-MSCs [36]. However, further analysis is necessary to fully confirm the ability of oPB-MSCs to differentiate into chondrocytes.

During the last decade, many reports have described the *in vitro* neural transdifferentiation of MSCs derived from a range of species [2,54,55] but, to our knowledge, this has never been investigated in oMSCs. Neurogenic capacity of PB-MSCs would offer exciting possibilities for autologous therapeutic treatments for a variety of neurological disorders. As ovine is a natural model for prion diseases, the transdifferentiation of MSCs into neural cells could provide an excellent *in vitro* model for the study of these pathologies. Here, we described alterations in the morphology and expression profiles of neurogenic markers (*MAP2, NEFM, NELF, NES* and *TUBB3*) that are consistent with neural differentiation. In addition, we detected up-regulation of *PRNP*, which could also be involved in the morphological changes as the cellular prion protein seems to be necessary for neuritogenesis [56]. The variable success in the ability to transdifferentiate MSCs to a neural phenotype could be influenced by the inter-donor variability of expression of neural-related markers in MSCs prior to differentiation [57]. Nevertheless, our study shows that oPB-MSCs retain the ability to transdifferentiate. Finally, although murine bone marrow stromal cells express the prion protein [58], this has not been previously shown in species susceptible to prion diseases. In the present work, we have demonstrated the expression of *PRNP* in oPB-MSCs and its overexpression during neuronal differentiation.

## Conclusions

In this study we describe, for the first time, the isolation of mesenchymal stem cells from ovine peripheral blood. These cells express mesenchymal markers and retain the ability to differentiate into adipocytes and osteoblasts. Although oPB-MSCs seem to differentiate into chondrocytes, further studies are necessary to confirm the suitability of these cells for chondrogenesis studies. Finally, these cells can transdifferentiate into neuron-like cells and express *PRNP*.

## Methods

### Animals and MSC isolation

Peripheral blood (25 mL) was obtained from a total of 6 sheep aged 1.5 to 6 years. The animals belonged to the Rasa Aragonesa breed and came from regional flocks. The procedure for blood collection from commercial farm animals was performed according to the recommendations of the Joint Working Group on Refinement [59]. The ethics committee of the University of Zaragoza approved the study (PI38/10). The blood was collected in 5 mL tubes with sodium heparin. Immediately after, blood was diluted in 1 volume of PBS and layered over Lymphoprep (Atom) in a 1:1 proportion. The mononuclear fraction was harvested after a density gradient centrifugation step of 20 min at 1600 g. Mononuclear cells were rinsed twice in the same volume of PBS by centrifugation for 5 min at 1600 g. The cells were resuspended, counted and plated at 10^6^ cells/cm^2^ in 6-well plates with basal medium consisting of low glucose Dulbecco’s modified Eagle’s medium (Sigma-Aldrich) supplemented with 20% foetal bovine serum (FBS), 1% L-glutamine (Sigma-Aldrich) and 1% streptomycin/penicillin (Sigma-Aldrich).

Non-adherent cells were removed washing the mononuclear cells twice with PBS after 24, 48 and 72 h of incubation at 37°C and 5% CO_2_ and were maintained in growth medium until reaching approximately 80% confluence. The cells were then treated with trypsin (Sigma Aldrich) and plated either in T75 or T175 flasks (Becton Dickinson) at 5000 cells/cm^2^ in basal medium with 10% FBS. The cells were trypsinised until the second passage (P2) and then cryopreserved in FBS with 10% DMSO.

The yield of adherent cells during these two passages was used to characterize the self renewal capacity of the cells isolated towards the estimation of the cell doubling (CD) and the doubling time (DT) parameters. These values were calculated using the formula: CD = ln (Nf/Ni)/ ln2; DT: time (days)/ CD, Nf being the final number of cells in the culture, and Ni the initial number.

Approximately 10^6^ cells from passage two were thawed at 37°C and plated in a T75 flask. Cells were grown for two more passages prior to being used for the differentiation analyses.

### Adipogenic differentiation

The cells obtained from the 6 sheep were seeded at 5000 cells/cm^2^ in 24-well plates with a previously described adipogenic medium [39]. Four replicates were seeded for each sheep, two were cultured with growth (control) medium and the other two with the adipogenic medium. The medium was changed every 3 days, and the differentiation was maintained for 14 days. To analyse the adipogenic differentiation, cells were fixed in 10% formalin (Sigma-Aldrich) for 15 min, and lipid droplets formed inside the cells were stained with 0.3% oil red O (Sigma-Aldrich). The expression of the adipogenic markers *PPARG*, *SCD* and *IL6* was analysed at days 7 and 14 of culture using RT-qPCR.

### Osteogenic differentiation

Cells from the 6 sheep were plated at 2 x 10^4^ cells/cm^2^ in 24-well plates and cultured under osteogenic conditions (two replicates) or with growth medium (two replicates) for 21 days as previously described [39]. To assess their osteogenic potential, cells at days 7, 14 and 21 were fixed in 70% ethanol for 1 h and stained with 2% Alizarin Red S (Sigma Aldrich) for 10 min. The transcript expression of the osteogenic markers *COL1A1* and *BGLAP* was evaluated by RT-qPCR at days 7, 14 and 21 of culture.

### Chondrogenic differentiation

For chondrogenic differentiation in monolayer cultures (n = 5), 10^5^ cells/cm^2^ were seeded in 24-well plates with the chondrogenic media described by Jäger et al. [53] (two replicates) or with growth medium (two replicates). The culture was maintained for 21 days with the media being changed twice per week. To determine chondrogenic differentiation, the cultures were stained with alcian blue dye (Sigma-Aldrich). Briefly, cells were washed with PBS, fixed with 70% ethanol for 1 h at room temperature, washed three times with distilled water, stained with alcian blue stain diluted in methanol at a 1:1 proportion and washed with water until the excess staining was removed. *BGN* and *LUM* transcripts were quantified at days 10 and 21 of chondrogenic induction using RT-qPCR.

### Neuronal differentiation

The neurogenic potential of the isolated cells was tested in two cell lines. The cells were seeded at 2500 cells/cm^2^ in 24-well plates with the neurogenic medium (Thermo Scientific) (two replicates) or under growth medium (two replicates) and maintained for 6 days, changing the media every 3 days. Differentiation was monitored by both light microscope and analysis of the mRNA expression levels of neurogenic markers (*MAP2*, *NELF*, *NES*, *NEFM* and *TUBB3*) and *PRNP* by RT-qPCR at days 3 and 6.

### Real Time quantitative PCR

The potential of cultured cells to differentiate into adipocytes, osteoblasts, chondrocytes and nervous cells was monitored via analysis of the expression levels of differentiation markers (Table 1) using RT-qPCR. The same methodology was used to evaluate the expression levels of cell surface markers for mesenchymal (*CD29*, *CD73*, *CD90* and *CD105*) and haematopoietic (*CD34* and *CD45*) stem cells in undifferentiated cells. The primers for RT-qPCR were designed using Primer Express 2.0 software (Applied Biosystems).

**Table 1:** Cell surface, adipogenic, osteogenic, chondrogenic and neurogenic markers analysed by RT-qPCR

**Genes**	**Accession number**	**Primer sequences**		**Amplicon size (bp)**
		**Forward (5’ → 3’)**	**Reverse (5’ → 3’)**	
***Cell Surface Markers***				
***CD29***	AF349461	GTGCCCGAGCCTTCAATAAAG	CCCGATTTTCAACCTTGGTAATG	87
***CD34***	AB021662	TGGGCATCGAGGACATCTCT	GATCAAGATGGCCAGCAGGAT	107
***CD45***	NM_001206523	CCTGGACACCACCTCAAAGCT	TCCGTCCTGGGTTTTATCCTG	101
***CD73***	BC114093	TGGTCCAGGCCTATGCTTTTG	GGGATGCTGCTGTTGAGAAGAA	115
***CD90***	BC104530	CAGAATACAGCTCCCGAACCAA	CACGTGTAGATCCCCTCATCCTT	96
***CD105***	NM_001076397	CGGACAGTGACCGTGAAGTTG	TGTTGTGGTTGGCCTCGATTA	115
	***Differentiation Markers***				
***PPARG***	NM_001100921	GCCCTGGCAAAGCATTTGTA	TGTCTGTCGTCTTTCCCGTCA	94	
***SCD***^***1***^	AJ001048	CCCAGCTGTCAGAGAAAAGG	GATGAAGCACAACAGCAGGA	115	
***IL6***	FJ409227.1	CAGCAAGGAGACACTGGCAG	TGATCAAGCAAATCGCCTGAT	101	
***COL1A1***	AF129287	CCTGCGTACAGAACGGCCT	ACAGCACGTTGCCGTTGTC	93	
***BGLAP***	DQ418490	CCCAGGAGGGAGGTGTGTG	CTAGACCGGGCCGTAGAAGC	99	
***BGN***	NM_001009201.1	AACATGAACTGCATTGAGATGGG	GCGAAGGTAGTTGAGCTTCAGG	93	
***LUM***	NM_173934.1	AAGCAATTGAAGAAGCTGCACA	TTAGTGAGCTGCAGGTCCACC	92	
***NES***	194665083	CAAATCGCCCAGGTCCTG	GCCTCTAGGAGGGTCCTGTATGT	95	
***NEFM***	194669578	GCTCGTCATCTGCGAGAATACC	CACCCTCCAGGAGTTTCCTGTA	91	
***NELF***	27806522	CGCTATGCAGGACACAATCAAC	GGGTCTCCTCACCTTCCAAGA	161	
***TUBB3***	116004470	GACCTCGAGCCTGGAACCAT	GCCCCACTCTGACCAAAGATG	92	
***MAP2***	194664873	TGTCCCAGTGGAGGAAGGTTT	TCTTGTCTAGTGGCTCGGCTG	95	
***PRNP***	BC119821	CGCAGAAGCAGGACTTCTGAA	TGGATTTGTGTCTCTGGGAAGA	86	
***Housekeeping genes***					
***G6PDH***^***2***^	AJ507200	TGACCTATGGCAACCGATACAA	CCGCAAAAGACATCCAGGAT	76	
***HPRT***^***3***^	EF078978	AGGTGTTTATTCCTCATGGAGTAATTATG	GGCCTCCCATCTCCTTCATC	79	

GenBank accession numbers of the sequences used for primer design. Primer sequences (F: Forward and R: Reverse) and the length of the amplicon in base pairs (bp).

^1^ Primers described in Dervishi et al. [60].

^2^ Primers described in García-Crespo et al. 2005 [61].

^3^ Primers described in Lyahyai et al. 2010 [62].

RNA extraction and cDNA synthesis were performed on both differentiated and control oPB-MSC cultures using the cells-to-cDNA kit (Ambion). The isolated cDNA was diluted 1:5 in water for further analysis. Amplification experiments were performed in triplicate using Fast SYBR Green Master Mix reagent (Life Technologies) and the StepOne™ Real Time System (Life Technologies). The levels of gene expression were determined using the comparative Ct method. A normalization factor (NF) calculated as the geometric mean of the quantity of two housekeeping genes (*GAPDH* and *HPRT*) was used to normalize the expression levels for each gene. Variations in gene expression between differentiated and control oPB-MSCs were evaluated with the Student’s *t* test. Statistical significance was defined as *P* < 0.05.

## Abbreviations

*BGLAP*: Bone Gamma-Carboxyglutamate (Gla) Protein; *BGN*: Biglycan; *CD105*: Endoglin; *CD29*: Integrin beta 1; *CD34*: CD34 molecule; *CD45*: Protein Tyrosine Phosphatase Receptor Type C; *CD73*: Ecto-5’-nucleotidase; *CD90*: Thy-1 cell surface antigen; *COL1A1*: *C*ollagen type I alpha 1; FBS: Foetal Bovine Serum *IL6,* interleukin 6 (interferon, beta 2); *LUM*: Lumican; *MAP2*: Microtubule-Associated Protein 2; MSCs: Mesenchymal Stem Cells; *NEFM*: Neurofilament, Medium Polypeptide; *NELF*: Nasal Embryonic LHRH Factor; *NES*: Nestin; oAT-MSCs: ovine Adipose Tissue-Derived Mesenchymal Stem Cells; oBM-MSCs: ovine Bone Marrow-Derived Mesenchymal Stem Cells; oPB-MSCs: ovine Peripheral Blood-derived Mesenchymal Stem Cells; oMSCs: ovine Mesenchymal Stem Cells; *PPARG*: Peroxisome Proliferator-Activated Receptor Gamma; *PRNP*: Prion Protein; RT-qPCR: quantitative Real Time PCR; *SCD*: Stearoyl-CoA Desaturase (delta-9-desaturase); *TUBB3*: Tubulin Beta 3.

## Competing interests

The authors declare that they have no competing interests.

## Authors’ contributions

JL and DRM carried out the isolation and expansion of the cells, differentiation assays, gene expression analyses, statistical analysis and participated in drafting the manuscript. BR, AS and ARR helped in the culture and differentiation assays and manuscript drafting. RB performed the sample collections from the animals and participated in cell isolation. PZ and CR helped to draft the manuscript. IMB conceived the study, participated in its design and draft the manuscript. All authors read and approved the final manuscript.

## References

[B1] Pittenger MF Mackay AM Beck SC Jaiswal RK Douglas R Mosca JD Moorman MA Simonetti DW Craig S Marshak DR Multilineage potential of adult human mesenchymal stem cells Science 1999 284 143 147 10.1126/science.284.5411.143 10102814

[B2] Bossolasco P Cova L Calzarossa C Rimoldi SG Borsotti C Deliliers GL Silani V Soligo D Polli E Neuro-glial differentiation of human bone marrow stem cells in vitro Exp Neurol 2005 193 312 325 10.1016/j.expneurol.2004.12.013 15869934

[B3] Gimble J Guilak F Adipose-derived adult stem cells: isolation, characterization, and differentiation potential Cytotherapy 2003 5 362 369 10.1080/14653240310003026 14578098

[B4] Caplan AI Review: mesenchymal stem cells: cell-based reconstructive therapy in orthopedics Tissue Eng 2005 11 1198 1211 10.1089/ten.2005.11.1198 16144456

[B5] Guo X Wang C Duan C Descamps M Zhao Q Dong L Lu S Anselme K Lu J Song YQ Repair of osteochondral defects with autologous chondrocytes seeded onto bioceramic scaffold in sheep Tissue Eng 2004 10 1830 1840 10.1089/ten.2004.10.1830 15684691

[B6] Scheerlinck JP Snibson KJ Bowles VM Sutton P Biomedical applications of sheep models: from asthma to vaccines Trends Biotechnol 2008 26 259 266 10.1016/j.tibtech.2008.02.002 18353472

[B7] Psaltis PJ Carbone A Nelson AJ Lau DH Jantzen T Manavis J Williams K Itescu S Sanders P Gronthos S Zannettino AC Worthley SG Reparative effects of allogeneic mesenchymal precursor cells delivered transendocardially in experimental nonischemic cardiomyopathy JACC Cardiovasc Interv 2010 3 974 983 10.1016/j.jcin.2010.05.016 20850099

[B8] Sill B Roy N Hammer PE Triedman JK Sigg DC Kelly MF Nedder A Dunning PS Cowan DB Development of an ovine model of pediatric complete heart block J Surg Res 2011 166 e103 e108 10.1016/j.jss.2010.11.878 21227467 PMC3073560

[B9] Fauza DO Jennings RW Teng YD Snyder EY Neural stem cell delivery to the spinal cord in an ovine model of fetal surgery for spina bifida Surgery 2008 144 367 373 10.1016/j.surg.2008.05.009 18707035

[B10] Hunter N Scrapie and experimental BSE in sheep Br Med Bull 2003 66 171 183 10.1093/bmb/66.1.171 14522858

[B11] Lyahyai J Bolea R Serrano C Monleon E Moreno C Osta R Zaragoza P Badiola JJ Martin-Burriel I Correlation between Bax overexpression and prion deposition in medulla oblongata from natural scrapie without evidence of apoptosis Acta Neuropathol 2006 112 451 460 10.1007/s00401-006-0094-4 16804709

[B12] Le Blanc K Pittenger M Mesenchymal stem cells: progress toward promise Cytotherapy 2005 7 36 45 10.1080/14653240510018118 16040382

[B13] Krampera M Marconi S Pasini A Galie M Rigotti G Mosna F Tinelli M Lovato L Anghileri E Andreini A Pizzolo G Sbarbati A Bonetti B Induction of neural-like differentiation in human mesenchymal stem cells derived from bone marrow, fat, spleen and thymus Bone 2007 40 382 390 10.1016/j.bone.2006.09.006 17049329

[B14] Fadel L Viana BR Feitosa ML Ercolin AC Roballo KC Casals JB Pieri NC Meirelles FV Martins Ddos S Miglino MA Ambrosio CE Protocols for obtainment and isolation of two mesenchymal stem cell sources in sheep Acta Cir Bras 2011 26 267 273 10.1590/S0102-86502011000400004 21808838

[B15] He Q Wan C Li G Concise review: multipotent mesenchymal stromal cells in blood Stem Cells 2007 25 69 77 10.1634/stemcells.2006-0335 16973831

[B16] Valenti MT Dalle Carbonare L Donatelli L Bertoldo F Zanatta M Lo Cascio V Gene expression analysis in osteoblastic differentiation from peripheral blood mesenchymal stem cells Bone 2008 43 1084 1092 10.1016/j.bone.2008.07.252 18761114

[B17] Koerner J Nesic D Romero JD Brehm W Mainil-Varlet P Grogan SP Equine peripheral blood-derived progenitors in comparison to bone marrow-derived mesenchymal stem cells Stem Cells 2006 24 1613 1619 10.1634/stemcells.2005-0264 16769763

[B18] Zvaifler NJ Marinova-Mutafchieva L Adams G Edwards CJ Moss J Burger JA Maini RN Mesenchymal precursor cells in the blood of normal individuals Arthritis Res 2000 2 477 488 10.1186/ar130 11056678 PMC17820

[B19] Kuwana M Okazaki Y Kodama H Izumi K Yasuoka H Ogawa Y Kawakami Y Ikeda Y Human circulating CD14+ monocytes as a source of progenitors that exhibit mesenchymal cell differentiation J Leukoc Biol 2003 74 833 845 10.1189/jlb.0403170 12960274

[B20] Rentsch C Hess R Rentsch B Hofmann A Manthey S Scharnweber D Biewener A Zwipp H Ovine bone marrow mesenchymal stem cells: isolation and characterization of the cells and their osteogenic differentiation potential on embroidered and surface-modified polycaprolactone-co-lactide scaffolds In Vitro Cell Dev Biol Anim 2010 46 624 634 10.1007/s11626-010-9316-0 20490706

[B21] McCarty RC Gronthos S Zannettino AC Foster BK Xian CJ Characterisation and developmental potential of ovine bone marrow derived mesenchymal stem cells J Cell Physiol 2009 219 324 333 10.1002/jcp.21670 19115243

[B22] Zscharnack M Poesel C Galle J Bader A Low oxygen expansion improves subsequent chondrogenesis of ovine bone-marrow-derived mesenchymal stem cells in collagen type I hydrogel Cells Tissues Organs 2009 190 81 93 10.1159/000178024 19033681

[B23] Niemeyer P Fechner K Milz S Richter W Suedkamp NP Mehlhorn AT Pearce S Kasten P Comparison of mesenchymal stem cells from bone marrow and adipose tissue for bone regeneration in a critical size defect of the sheep tibia and the influence of platelet-rich plasma Biomaterials 2010 31 3572 3579 10.1016/j.biomaterials.2010.01.085 20153047

[B24] Kunisaki SM Fuchs JR Steigman SA Fauza DO A comparative analysis of cartilage engineered from different perinatal mesenchymal progenitor cells Tissue Eng 2007 13 2633 2644 10.1089/ten.2006.0407 17655491

[B25] Martinez-Lorenzo MJ Royo-Canas M Alegre-Aguaron E Desportes P Castiella T Garcia-Alvarez F Larrad L Phenotype and chondrogenic differentiation of mesenchymal cells from adipose tissue of different species J Orthop Res 2009 27 1499 1507 10.1002/jor.20898 19408284

[B26] Rhodes NP Srivastava JK Smith RF Longinotti C Heterogeneity in proliferative potential of ovine mesenchymal stem cell colonies J Mater Sci Mater Med 2004 15 397 402 15332606 10.1023/b:jmsm.0000021109.21807.f0

[B27] Dominici M Le Blanc K Mueller I Slaper-Cortenbach I Marini F Krause D Deans R Keating A Prockop D Horwitz E Minimal criteria for defining multipotent mesenchymal stromal cells The International Society for Cellular Therapy position statement Cytotherapy 2006 8 315 317 10.1080/14653240600855905 16923606

[B28] Wang X Moutsoglou D Osteogenic and adipogenic differentiation potential of an immortalized fibroblast-like cell line derived from porcine peripheral blood In Vitro Cell Dev Biol Anim 2009 45 584 591 10.1007/s11626-009-9231-4 19633899

[B29] Spaas JH Schauwer CD Cornillie P Meyer E Soom AV Van de Walle GR Culture and characterisation of equine peripheral blood mesenchymal stromal cells Vet J 2012 in press 10.1016/j.tvjl.2012.05.006 22717781

[B30] Wang H Pang B Li Y Zhu D Pang T Liu Y Dexamethasone has variable effects on mesenchymal stromal cells Cytotherapy 2012 14 423 430 10.3109/14653249.2011.652735 22364108

[B31] Radcliffe CH Flaminio MJ Fortier LA Temporal analysis of equine bone marrow aspirate during establishment of putative mesenchymal progenitor cell populations Stem Cells Dev 2010 19 269 282 10.1089/scd.2009.0091 19604071 PMC3138180

[B32] Rallapalli S Bishi DK Verma RS Cherian KM Guhathakurta S A multiplex PCR technique to characterize human bone marrow derived mesenchymal stem cells Biotechnol Lett 2009 31 1843 1850 10.1007/s10529-009-0106-2 19693443

[B33] Chong PP Selvaratnam L Abbas AA Kamarul T Human peripheral blood derived mesenchymal stem cells demonstrate similar characteristics and chondrogenic differentiation potential to bone marrow derived mesenchymal stem cells J Orthop Res 2012 30 634 642 10.1002/jor.21556 21922534

[B34] Kassis I Zangi L Rivkin R Levdansky L Samuel S Marx G Gorodetsky R Isolation of mesenchymal stem cells from G-CSF-mobilized human peripheral blood using fibrin microbeads Bone Marrow Transplant 2006 37 967 976 10.1038/sj.bmt.1705358 16670702

[B35] Tondreau T Meuleman N Delforge A Dejeneffe M Leroy R Massy M Mortier C Bron D Lagneaux L Mesenchymal stem cells derived from CD133-positive cells in mobilized peripheral blood and cord blood: proliferation, Oct4 expression, and plasticity Stem Cells 2005 23 1105 1112 10.1634/stemcells.2004-0330 15955825

[B36] Mrugala D Bony C Neves N Caillot L Fabre S Moukoko D Jorgensen C Noel D Phenotypic and functional characterisation of ovine mesenchymal stem cells: application to a cartilage defect model Ann Rheum Dis 2008 67 288 295 17644536 10.1136/ard.2007.076620

[B37] Bian ZY Li G Gan YK Hao YQ Xu WT Tang TT Increased number of mesenchymal stem cell-like cells in peripheral blood of patients with bone sarcomas Arch Med Res 2009 40 163 168 10.1016/j.arcmed.2009.01.002 19427966

[B38] Ranera B Lyahyai J Romero A Vazquez FJ Remacha AR Bernal ML Zaragoza P Rodellar C Martin-Burriel I Immunophenotype and gene expression profiles of cell surface markers of mesenchymal stem cells derived from equine bone marrow and adipose tissue Vet Immunol Immunopathol 2011 144 147 154 10.1016/j.vetimm.2011.06.033 21782255

[B39] Ranera B Ordovás L Lyahyai J Bernal ML Fernandes F Romero A Vázquez FJ Osta R Cons C Varona L Zaragoza P Martín-Burriel I Rodellar C Comparative study of equine bone marrow- and adipose tissue-derived mesenchymal stem cells Equine Vet J 2012 44 33 42 10.1111/j.2042-3306.2010.00353.x 21668489

[B40] Seo JB Moon HM Kim WS Lee YS Jeong HW Yoo EJ Ham J Kang H Park MG Steffensen KR Stulnig TM Gustafsson JA Park SD Kim JB Activated liver X receptors stimulate adipocyte differentiation through induction of peroxisome proliferator-activated receptor gamma expression Mol Cell Biol 2004 24 3430 3444 10.1128/MCB.24.8.3430-3444.2004 15060163 PMC381668

[B41] Aguilar V Annicotte JS Escote X Vendrell J Langin D Fajas L Cyclin G2 regulates adipogenesis through PPAR gamma coactivation Endocrinology 2010 151 5247 5254 10.1210/en.2010-0461 20844002 PMC3000854

[B42] Menssen A Haupl T Sittinger M Delorme B Charbord P Ringe J Differential gene expression profiling of human bone marrow-derived mesenchymal stem cells during adipogenic development BMC Genomics 2011 12 461 10.1186/1471-2164-12-461 21943323 PMC3222637

[B43] Kim YC Ntambi JM Regulation of stearoyl-CoA desaturase genes: role in cellular metabolism and preadipocyte differentiation Biochem Biophys Res Commun 1999 266 1 4 10.1006/bbrc.1999.1704 10581155

[B44] Pricola KL Kuhn NZ Haleem-Smith H Song Y Tuan RS Interleukin-6 maintains bone marrow-derived mesenchymal stem cell stemness by an ERK1/2-dependent mechanism J Cell Biochem 2009 108 577 588 10.1002/jcb.22289 19650110 PMC2774119

[B45] Song L Webb NE Song Y Tuan RS Identification and functional analysis of candidate genes regulating mesenchymal stem cell self-renewal and multipotency Stem Cells 2006 24 1707 1718 10.1634/stemcells.2005-0604 16574750

[B46] Gronthos S Mrozik K Shi S Bartold PM Ovine periodontal ligament stem cells: isolation, characterization, and differentiation potential Calcif Tissue Int 2006 79 310 317 10.1007/s00223-006-0040-4 17033723

[B47] Jikko A Harris SE Chen D Mendrick DL Damsky CH Collagen integrin receptors regulate early osteoblast differentiation induced by BMP-2 J Bone Miner Res 1999 14 1075 1083 10.1359/jbmr.1999.14.7.1075 10404007

[B48] Liu F Akiyama Y Tai S Maruyama K Kawaguchi Y Muramatsu K Yamaguchi K Changes in the expression of CD106, osteogenic genes, and transcription factors involved in the osteogenic differentiation of human bone marrow mesenchymal stem cells J Bone Miner Metab 2008 26 312 320 10.1007/s00774-007-0842-0 18600396

[B49] Zou L Zou X Chen L Li H Mygind T Kassem M Bunger C Multilineage differentiation of porcine bone marrow stromal cells associated with specific gene expression pattern J Orthop Res 2008 26 56 64 10.1002/jor.20467 17676606

[B50] Sollazzo V Palmieri A Scapoli L Martinelli M Girardi A Pellati A Scarano A Perrotti V Spinelli G Carinci F Allogro® acts on stem cells derived from peripheral blood The Internet Journal of Dental Science 2009 8

[B51] Aubin JE Bone stem cells J Cell Biochem Suppl 1998 30-31 73 82 9893258

[B52] Zscharnack M Hepp P Richter R Aigner T Schulz R Somerson J Josten C Bader A Marquass B Repair of chronic osteochondral defects using predifferentiated mesenchymal stem cells in an ovine model Am J Sports Med 2010 38 1857 1869 10.1177/0363546510365296 20508078

[B53] Jager M Bachmann R Scharfstadt A Krauspe R Ovine cord blood accommodates multipotent mesenchymal progenitor cells In Vivo 2006 20 205 214 16634520

[B54] Woodbury D Schwarz EJ Prockop DJ Black IB Adult rat and human bone marrow stromal cells differentiate into neurons J Neurosci Res 2000 61 364 370 10.1002/1097-4547(20000815)61:4<364::AID-JNR2>3.0.CO;2-C 10931522

[B55] Jori FP Napolitano MA Melone MA Cipollaro M Cascino A Altucci L Peluso G Giordano A Galderisi U Molecular pathways involved in neural in vitro differentiation of marrow stromal stem cells J Cell Biochem 2005 94 645 655 10.1002/jcb.20315 15547939

[B56] Loubet D Dakowski C Pietri M Pradines E Bernard S Callebert J Ardila-Osorio H Mouillet-Richard S Launay JM Kellermann O Schneider B Neuritogenesis: the prion protein controls beta1 integrin signaling activity FASEB J 2012 26 678 690 10.1096/fj.11-185579 22038049

[B57] Montzka K Lassonczyk N Tschoke B Neuss S Fuhrmann T Franzen R Smeets R Brook GA Woltje M Neural differentiation potential of human bone marrow-derived mesenchymal stromal cells: misleading marker gene expression BMC Neurosci 2009 10 16 10.1186/1471-2202-10-16 19257891 PMC2655300

[B58] Takakura Y Yamaguchi N Nakagaki T Satoh K Kira J Nishida N Bone marrow stroma cells are susceptible to prion infection Biochem Biophys Res Commun 2008 377 957 961 10.1016/j.bbrc.2008.10.099 18976632

[B59] Joint Working Group on Refinement Removal of blood from laboratory mammals and birds. First report of the BVA/FRAME/RSPCA/UFAW Joint Working Group on Refinement Lab Anim 1993 27 1 22 8437430 10.1258/002367793781082412

[B60] Dervishi E Serrano C Joy M Serrano M Rodellar C Calvo JH Effect of the feeding system on the fatty acid composition, expression of the Delta9-desaturase, Peroxisome Proliferator-Activated Receptor Alpha, Gamma, and Sterol Regulatory Element Binding Protein 1 genes in the semitendinous muscle of light lambs of the Rasa Aragonesa breed BMC Vet Res 2010 6 40 10.1186/1746-6148-6-40 20649987 PMC2917425

[B61] Garcia-Crespo D Juste RA Hurtado A Selection of ovine housekeeping genes for normalisation by real-time RT-PCR; analysis of PrP gene expression and genetic susceptibility to scrapie BMC Vet Res 2005 1 3 10.1186/1746-6148-1-3 16188044 PMC1262732

[B62] Lyahyai J Serrano C Ranera B Badiola JJ Zaragoza P Martin-Burriel I Effect of scrapie on the stability of housekeeping genes Anim Biotechnol 2010 21 1 13 20024782 10.1080/10495390903323851

